# A Liquid Chromatography-Tandem Mass Spectrometry Method for Evaluation of Two Brands of Enalapril 20 mg Tablets in Healthy Human Volunteers

**DOI:** 10.1155/2017/8489471

**Published:** 2017-08-15

**Authors:** Wael Abu Dayyih, Mohammed Hamad, Ahmad Abu Awwad, Eyad Mallah, Zainab Zakarya, Alice Abu Dayyih, Tawfiq Arafat

**Affiliations:** ^1^Faculty of Pharmacy and Medical Sciences, University of Petra, Amman, Jordan; ^2^Department of Basic Sciences, College of Science and Health Professions, King Saud Bin Abdulaziz University for Health Sciences, Jeddah, Saudi Arabia; ^3^Faculty of Chemistry, University of Duisburg-Essen, Essen, Germany; ^4^School of Chemistry, Biology and Pharmacy, Hochschule Fresenius University of Applied Sciences, Idstein, Germany

## Abstract

Enalapril is an angiotensin-converting enzyme inhibitor used for treatment of hypertension and chronic heart disease. Enalaprilat is its active metabolite responsible for the activity. This study aimed to develop and validate a method for enalapril and enalaprilat analysis and to determine the bioequivalence of two tablet formulae of enalapril. LC-MS/MS bioanalytical method was developed and validated and then applied to evaluate the bioavailability of two enalapril formulae. Antihyperglycemic sitagliptin was used as internal standard (IS). The method was accurate for the within- and between-days analysis, and precise CV% was <5%, being linear over the calibration range 1.0–200.0 ng/ml. Stability was >85% and the LOD was 0.907 and 0.910 ng/ml for enalapril and enalaprilat, respectively, and LLOQ was 1 ng/ml. The pharmacokinetic parameters *C*_max_, *t*_max_, AUC_0–72_, and AUC_0–*∞*_ values of enalapril and enalaprilat of the two formulae were calculated and nonsignificant differences were found. A linearity, specific, accurate, and precise method was developed and applied for the analysis of enalapril and enalaprilat in human plasma after oral administration of two formulae of enalapril 20 mg tablets in healthy volunteers. Depending on the statistical analysis it was concluded that the two enalapril formulae were bioequivalent.

## 1. Introduction

Hypertension and cardiovascular diseases are of the major causes of morbidity in the world. Antihypertensive drugs are a class of drugs that are used for treating hypertension (high blood pressure) and cardiovascular disease.

### 1.1. Enalapril and Enalaprilat

**Figure 1:**
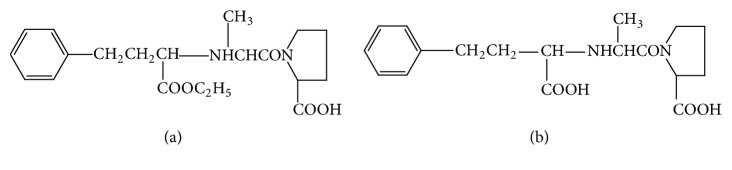
Structural formulae of (a) enalapril and (b) enalaprilat.

Enalapril maleate is chemically described as [(S)-1-(N-(1-(ethoxycarbonyl)-3-phenylpropyl)-L-alanyl)-Lproline (Z)-2-butenedioate salt], and its empirical formula is (C_20_H_28_N_2_O_5_-C_4_H_4_O_4_) (Figure 1(a)). It is an angiotensin-converting enzyme (ACE) inhibitor, used for oral treatment of hypertension. It prevents the conversion of angiotensin I into angiotensin II mediated by ACE and the inactivation of kinins, resulting in decreased levels of vasoconstrictor peptide angiotensin II and the accumulation of kinins [1]. In addition, this agent plays a role in decreasing the mortality and morbidity rats of cardiovascular and heart failure patients [2].

Enalapril is a prodrug that is hydrolyzed (in vivo deesterification) after absorption in liver producing enalaprilat, the active angiotensin-converting enzyme (ACE) inhibitor (Figure 1(b)). Enalaprilat is the main metabolite of enalapril and it is more potent in the treatment of hypertension and congestive heart failure [3, 4]. It acts as a competitive inhibitor for ACE preventing conversion of angiotensin I to angiotensin II which acts as a vasoconstrictor material and stimulates the secretion of aldosterone. The binding of the enalaprilat to the enzyme forms a complex with slow dissociation rate which leads to high influence and long period of action [5].

Enalapril reaches its maximum plasma concentration within 1 hour after oral administration [3, 4]. It is rapidly absorbed from the gastrointestinal (GI) tract and the bioavailability is about 60–70%. The terminal half-life of enalapril is about 2 h and enalaprilat half-life of approximately 30–35 hrs [3, 4, 6]. The absorption of enalapril is not affected by the presence of food in the GI tract. However, its level did not reach 10 ng/ml after 4 hrs, while enalaprilat maximum plasma concentration was detected in about 3-4 hrs after administration [3, 4, 6].

### 1.2. Sitagliptin

**Figure 2:**
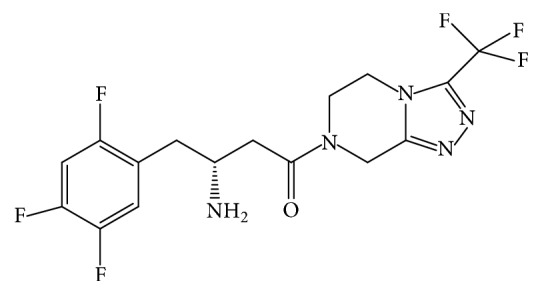
Structural formulae of sitagliptin.

Sitagliptin phosphate salt is an oral antihyperglycemic (antidiabetic) drug (Figure 2). It acts as dipeptidyl peptidase-4 (DPP-4) inhibitor. This drug is used either alone or in combination with other oral antihyperglycemic agents (such as metformin or a thiazolidinedione) for treatment of diabetes mellitus type 2 [7]. The DDP-4 inhibitors decrease blood glucose level by deactivating DPP-4, an enzyme responsible for breaking down the gastrointestinal hormone glucagon-like peptide-1 (GLP-1). The prevention of GLP-1 inactivation will increase the secretion of insulin in response to high level of blood glucose.

A meta-analysis of 18 phase III randomized controlled trials stated that DDP-4 inhibitors were related to a 52% (95% confidence interval 0.31% to 0.75%) qualified reduction in the adverse cardiovascular events (acute coronary syndrome, stroke, arrhythmias, heart failure or cardiovascular death, and nonfatal myocardial infarction) in comparison to other active or placebo treatments [8]. In addition, a recent study showed that sitagliptin use did not affect the risk for heart failure in hospitalizing type 2 diabetes mellitus patients, both overall and among high-risk patient subgroups [9].

Several bioanalytical methods were applied for the simultaneous quantification of enalapril and enalaprilat in human plasma using both high performance liquid chromatography (HPLC) and mass spectrometry (MS). One study using LC-MS method reported that after oral administration of enalapril the mean value of maximum plasma concentration (*C*_max_) is 102 ± 39 ng/ml and plasma time half-life (*t*_max_) is 0.96 ± 0.36 h [10]. Another study using a GC-MS method reported that after single oral intake of 20 mg of enalapril by 24 healthy subjects the mean value of *C*_max_ is 72.9 ± 41.6 ng/ml and *t*_max_ is 4.0 ± 1.4 h [11]. In addition, a study conducted by Lu et al. [12] using LC-MS/MS method revealed that (*C*_max_) were 90.5 ± 28.4 and 47.5 ± 12.4 ng/ml, *t*_max_ were 0.860 ± 0.310 and 4.20 ± 1.06 h, AUC_0–*t*_ was 136 ± 36 and 401 ± 89 ng/ml h, AUC_0–*∞*_ were 138 ± 36 and 420 ± 91 ng/ml h, and* T*1/2 were 1.35 ± 0.61 and 6.71 ± 2.22 h for enalapril and enalaprilat, respectively [12].

This study aimed to develop and validate a method using LC-MS/MS to measure enalapril and enalaprilat in plasma samples and to determine the bioequivalence of a new tablet formulation of enalapril (20 mg tablets).

## 2. Materials and Methods

### 2.1. Materials

#### 2.1.1. Reagents

Reagents were as follows: Deionized Water, Nanopure (Fisher Scientific); methanol advanced gradient grade (Fisher Scientific); formic acid (GPR, Rectapur); acetonitrile (ACN) (Fisher Scientific); enalapril and sitagliptin raw material (were a kind gift from Dar al Dawa Pharma, Jordan). The blank plasma sample was obtained from donor's blood treated with sodium heparin.

#### 2.1.2. Instrumentation

An API 1400 mass spectrometer was used, protected by a built-in waste/detector switching valve (AB Sciex, Framingham, MA, USA) and high performance liquid chromatography (HPLC) system (Agilent Technologies, model LC-1200, Englewood, USA) equipped by an autosampler and controlled by Analyst 1.6.1 software. Windows XP, SP3 Data Management Software 1.5.2 and, also, Bath Sonicator Crest model-175T (Ultra Sonics CORP) and Sartorius balance BP 2215 and Centrifuge (Eppendorf 5417C) were used.

#### 2.1.3. Chromatographic Conditions

The chromatographic conditions of LC-MS/MS were improved quantitatively for maximum analytical peak quality with short run time, using a mobile phase prepared by mixing of 70% methanol and 30% of a mixture of 20 mM ammonium acetate and 0.2 mM formic acid and was eluted isocratically with a flow rate of 1 mL min^−1^ through ACE™ C18 (50 × 2.1 mm, 5 *μ*m) column for LC-MS/MS. The injection volumes were 2 *μ*L. The MS parameters were improved for enalapril and enalaprilat analysis in ESI negative mode as follows: nitrogen gas 1 flow = 55 units, gas 2 = 70 units, curtain gas = 30 units, ion spray voltage = 5000 V, drying temperature = 550°C, and collision energy of 20 V.

### 2.2. Methods

#### 2.2.1. Preparation of Stock Solutions and Working Standards

The enalapril and enalaprilat assay standards were prepared by dissolving 1 mg of each in 1 mL of dH_2_O and then diluted in drug-free pooled heparinized human plasma to prepare a stock solution of 200 *μ*g/mL. From that stock solution, 200, 120, 50, 100, 15, 5, 2, 1, and 0 ng/mL of enalapril and enalaprilat standards and 1 ng/ml (LLOQ), 3 ng/ml (low), 100 ng/ml (medium), and 160 ng/ml (high) for QC samples were prepared. All stock solutions (1.0 mg/ml) and QC solutions were stored at −20°C and working standard solutions were stored at 4°C. The working internal standard solution (sitagliptin 500 ng/ml) was prepared in acetonitrile by taking 50 mg in a 100 ml volumetric flask being then dissolved in 10 ml acetonitrile. Then volume was made up to the mark with acetonitrile to obtain a solution containing 500 *μ*g/ml sitagliptin.

#### 2.2.2. Sample Preparation and Extraction

Sample preparation was performed by using one-step protein precipitation with acetonitrile. After thorough mixing of samples, 300 *μ*l plasma from each calibration standard, QC, and volunteers samples was pipetted in 1 ml centrifuge tube and then 900 *μ*l acetonitrile containing 500 ng/ml sitagliptin (IS) was added and followed by mixing for 2 min by vortex and centrifugation at 3,000 rpm for 10 min. Then organic layer was separated and taken into autosampler vials for LC-MS/MS analysis.

#### 2.2.3. Analytical Methods

Enalapril, enalaprilat levels in plasma, and the internal standard sitagliptin were measured by API 4000 LC-MS/MS. Protein precipitation were done for each sample by using acetonitrile. The mobile phase was prepared by mixing of 70% methanol and 30% of a mixture of 20 mM ammonium acetate and 0.2 mM formic acid, while the stationary phase was RP-C18 column (50 × 4.0 mm ID, 5 *μ*m). The column was maintained at 40°C, while the autosampler temperature was 4°C. The flow rate was 1.0 ml/min. The plasma levels of the analytes were measured after a single dose of 20 mg enalapril tablets under fasting conditions.

#### 2.2.4. Method Validation

In order to demonstrate the reliability of our method, accuracy, precision, linearity, and stability tests were carried out per EMEA 2012 and FA guidelines on bioanalytical method validation [13, 14] and also we followed the method developed in [15]. Regarding linearity test, 7 calibration points (1, 2, 5, 15, 100, 120, and 200 ng/ml) were involved, a series of 6 injections to each calibration concentration level. Peak areas of the calibration standards were plotted in the *y*-axis against the nominal standard concentration, and the linearity of the plotted curve was evaluated via calculation of the correlation coefficient (*R*^2^) which should be more than 0.98. Samples were injected to LC-MS/MS, to eliminate human error factor in sample preparation.

*(1) Specificity and LLOQ*. Specificity was defined as lacking of interference of enalapril and enalaprilat at retention times from the plasma components and lack of cross-interference between analytes and IS using extraction method and LC-MS/MS. Specificity of the method was assessed with LC-MS/MS. Six plasma samples were assessed in replicate analysis (*n* = 6) and assessed for interfering with contrast to LLOQ. Also, carryover was assessed by injecting blank samples after calibration standard sample at the upper limit of quantification.

*(2) Accuracy Precision and Linearity*.  Table 1 shows the results of accuracies and precisions measurement for intra- and interday runs for all analytes. The within-run accuracy and precision values were evaluated for LC-MS/MS by running analytical batch containing replicates (*n* = 6) from LLOQ and each QC level with calibration curve. Between-runs linearity, accuracy, and precision were evaluated by running three sets of within-run batches in three separate days.

*(3) Recovery and Matrix Effect*. The recovery study of enalapril and enalaprilat from protein precipitation procedure was assessed by using the low, medium, and high QC levels and comparing their peak areas with peak area in unprocessed samples (spiked blank with true corresponding amount). For enalapril, enalaprilat, and IS, the matrix factor (MF) was assessed individually from 6 different plasma sources by calculating the ratio of the peak area in presence of matrix (spiked enalapril and enalaprilat in blank's supernatant) to the peak area in absence of matrix (pure solution for enalapril and enalaprilat). IS normalized MF was also calculated by dividing the MF of enalapril and enalaprilat over MF of IS.

*(4) Stability*. Stability validation was done in triplicate run for low, mid, and high QC samples and calculated from spiked calibration curve. The spiked plasma and stock solution were kept at room temperature for 10 hrs to study the short-term stability, and then after 6 months another examination was performed for accelerated storage under −30°C. Three freeze-thaw cycles were applied to evaluate the freeze-thaw cycles stability for enalapril and enalaprilat in plasma by thawing from frozen state at room temperature for 1 hr and refreezing again for 24 hrs. The postextraction stability of the extracted sample was evaluated by injected samples after 48 h under autosampler cooling condition (4°C).

### 2.3. Clinical Study Design

In this study we applied a clinical study similar to that of [16] with modification. Thirty healthy adult male volunteers participated in this study after signing an informed consent. Volunteers were selected upon inclusion/exclusion criteria. Volunteers were enrolled in this study after obtaining their medical history and biochemical, serological, hematological and urine analysis and they were found healthy since there were no significant abnormalities in their results. Exclusion criteria included any medical history of a serious gastrointestinal tract problem which could affect the absorption of the drug and any history of allergy to fluoroquinolone derivatives. In addition, subjects who use chronic medication, such as theophylline, antacids, iron, or vitamins were excluded.

Before starting the study, volunteers were informed about the nature, purpose, duration, and risk of the study. Also, they were informed that they can withdraw from the study at any time. The study protocol was approved by the local institutional review board (number JCPR337/2012) and was in accordance with the Declaration of Helsinki (1964) as revised in Tokyo (1975), Venice (1983), Hong Kong (1989), and Somerset West, RSA (1996). Volunteers were also informed not to take any medication and alcohol for at least 1 week before starting the study. In addition, they were fasted for 12 hrs with free access to water before starting of the study. They stayed at the hospital during the whole period of the study.

#### 2.3.1. Application to a Bioequivalence Study

LC-MS/MS assay method was performed to compare the bioavailability of two formulae of enalapril (20 mg tablet) by applying a single oral dose, open label, randomized, two-period, two-sequence, crossover study of 30 healthy male volunteers under fasting condition. After fasting period (12 hrs, overnight), each volunteer was given an oral dose containing 20 mg of enalapril either “A, reference” or “B, tested” formula with 200 mL of water in the sequence determined by randomization. A wash-out period of 14 days had been applied between the two periods of the study.

The pharmacokinetic parameters considered for assessment of the bioequivalence study between the reference drug and test drug were as follows: maximum plasma concentration (*C*_max_), area under plasma concentration/time plot until the last quantifiable value (AUC_0–72_), auxiliary parameters, time of the maximum plasma concentration (*t*_max_), elimination half-life time (*t*-half), and area under plasma concentration/time plot extrapolated to infinity (AUC_0–*∞*_). Analysis of variance was achieved on the pharmacokinetic parameters. The 90% CIs of the pharmacokinetic parameters of the tested/reference drugs were calculated. All the pharmacokinetic parameters were determined by WinNonlin software (version 6.3) from Pharsight, USA.

#### 2.3.2. Blood Sampling

The median cubital vein was used to collect 5 mL venous blood samples before and after drug administration at “0.25, 0.50, 0.75, 1.00, 1.33, 1.66, 2.00, 2.50, 3.00, 3.50, 4.00, 4.50, 5.00, 6.00, 8.00, 10.00, 12.00, 24.00, 48.0, and 72.00” hours. Blood samples were withdrawn in labeled tubes containing lithium heparin and centrifuged at room temperature at 4000 rpm for 10 minutes. Plasma samples were taken and stored at −40°C till analysis.

## 3. Results

Enalapril was well tolerated by all volunteers; all volunteers completed the study and were discharged in good health. There was no drop-out of any volunteer.

### 3.1. Specificity and LLOQ

The LOD in plasma was found to be 0.907 and 0.910 ng/ml for both enalapril and enalaprilat, respectively, and LLOQ was 1 ng/ml in plasma.

### 3.2. Intra- and Interday Accuracy and Precision

The within-day and between-days accuracy range for both enalapril and enalaprilat were from 94 to 101% and 95 to 99%, respectively, for individual measurements in both while precision values (CV%) for enalapril and enalaprilat were less than 5%.  Table 1 summarizes the accuracy and precision values that resulted from the LC-MS/MS. The results showed that the used method has acceptable accuracy, precision, and reproducibility for the quantification of both enalapril and enalaprilat in human plasma. The method is accurate and precise as per the requirements of the FDA and EMA guidelines [13, 14]. The detected result verified a good linearity over the range 1–200 ng/ml for both enalapril and enalaprilat in human plasma.

### 3.3. Standard Calibration Curve Linearity

**Figure 3:**
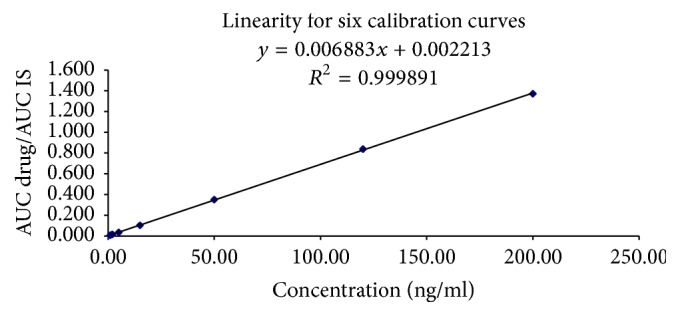
Calibration curve of peak area versus concentration (ng/ml) for enalapril.

**Figure 4:**
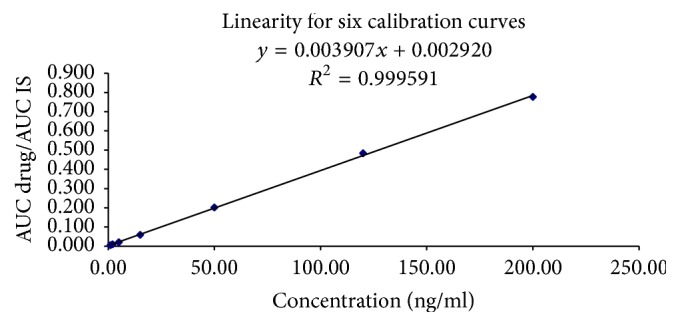
Calibration curve of peak area versus concentration (ng/ml) for enalaprilat.

The peak area ratios for enalapril and enalaprilat to IS in human plasma were linear over the calibration range from 1.0 to 200 ng/ml during the validation and the routine analysis for both enalapril (Figure 3) and enalaprilat (Figure 4) LC-MS/MS.  Table 2 shows the summary of calibration regression function parameters during method validation by using LC-MS/MS.

### 3.4. Recovery and Matrix Effect

The extraction procedures for enalapril and enalaprilat were highly efficient from plasma matrix and the recovery values for low OC, mid QC, and high QC for enalapril were 94.220%, 103.62%, and 101.96%, respectively, and those for enalaprilat were 98.17% for low QC, 108.40% for mid QC, and 101.83% for high QC (Table 3). No significant matrix effect was detected when comparing the AUC ratios of the extracted QCs and IS with the AUC of unextracted QCs and IS achieved from the injecting of raw solution prepared at the same levels.

### 3.5. Stability

The stability test results were greater than 85% for the spiked plasma and stock solutions kept under room temperature for 20 hrs, freeze-thaw cycles, autosampler, and accelerated stability for both enalapril and enalaprilat (Tables 4 and 5, resp.). Generally, the enalapril, enalaprilat, and IS were found stable under the different testing conditions with acceptable percent accuracy. Results were within the acceptance standards of European and US guideline for bioanalytical methods validation [13, 14].

### 3.6. Bioequivalence Study

**Figure 5:**
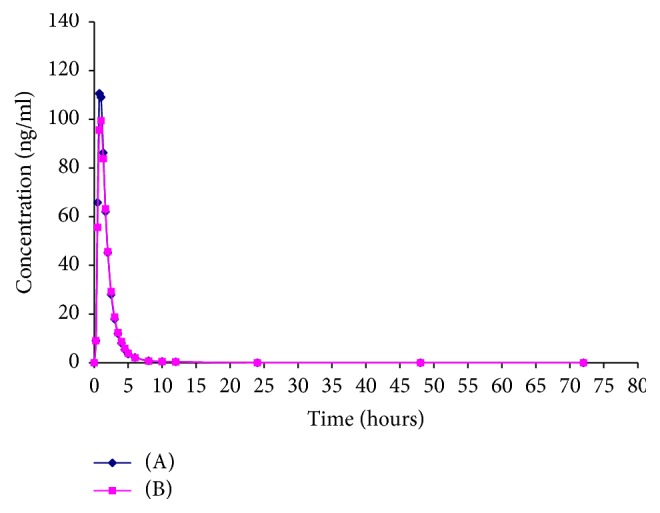
Graphical representation of mean plasma concentration versus time of enalapril following 20 mg single oral dose of test (B) and reference preparation (A) to 30 healthy human volunteers.

**Figure 6:**
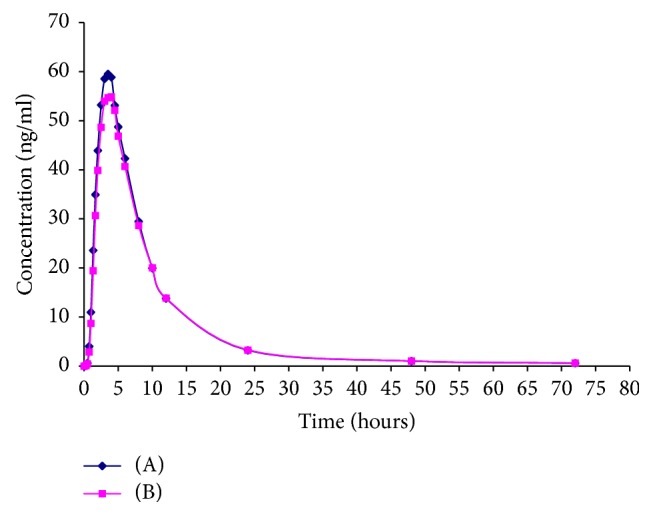
Graphical representation of mean plasma concentration versus time of enalaprilat following 20 mg single oral dose of test (B) and reference preparation (A) to 30 healthy human volunteers.

The mean plasma concentration-time profiles after single oral dose administration of reference and test formulations of enalapril 20 mg are shown in Figure 5 for enalapril and Figure 6 for enalaprilat. Figures show that the mean plasma concentration-time curves from the test and reference formulae are almost superimposable for both enalapril and enalaprilat. Furthermore, the mean estimated pharmacokinetic parameters calculated from the plasma concentration profiles are showing in Table 4. The mean maximum plasma concentrations (*C*_max_) of enalapril were 114.42 ± 43.75 and 121.18 ± 50.23 ng/ml for test and reference formulae, respectively, and for enalaprilat were 57.67 ± 17.09 and 62.24 ± 22.14 ng/ml, respectively. The time required to reach the maximum serum concentration (*t*_max_) of enalapril was 0.921 ± 0.308 for test and 0.897 ± 0.239 h for reference formulae and for enalaprilat was 3.667 ± 0.592 and 3.539 ± 0.639 hrs, respectively. Other parameters are found in Table 6.

Statistically no significant differences were found between any parameters of both formulations. The 90% CI values and mean log-transformed ratios for all parameters were found to be within the required limits confirming the bioequivalence of the test and reference formulations.

## 4. Conclusions

The developed bioanalytical method is valid, precise, accurate, stable, and robust. In addition, this method is selective, accurate, highly sensitive, and validated per EMA and FDA guidelines. It was used for analysis of enalapril, enalaprilat, and sitagliptin samples from a bioequivalence study. The statistical assessment of the pharmacokinetic parameters for enalapril and enalaprilat indicated that there is no significant difference between the test and reference formulations. The confidence intervals for the ratio of mean AUC_0–*t*_, AUC_0–*∞*_, and *C*_max_ showed that these parameters are within the bioequivalence acceptance range (using log-transformed data). The two formulations are equivalent and medically considered equal.

## Figures and Tables

**Table 1:** Intra- and interday precision and accuracy data for enalapril and enalaprilat in human plasma (*n* = 6).

	Intraday (within run)				Interday (between runs)			
	Mean concentration (ng/ml)	SD	CV (%)	Accuracy (%)	Mean concentration (ng/ml)	SD	CV (%)	Accuracy (%)
*Enalapril*								
LQC (3 ng/ml)	2.834	0.107	3.773	94.458	2.98	0.21		99.27
MQC (100 ng/ml)	100.691	4.08	4.05	100.691	100.68	4.93		100.68
HQC (1600 ng/ml)	161.366	7.169	4.459	100.854	162.48	12.62		101.55

*Enalaprilat*								
LQC (3 ng/ml)	2.876	0.124	4.295	95.861	3.310	0.210		110.34
MQC (100 ng/ml)	98.514	3.816	3.873	98.514	102.224	4.280		102.24
HQC (160 ng/ml)	152.412	4.517	2.964	95.257	162.11	11.43		101.32

LQC: low quality control; MQC: medium quality control; HQC: high quality control.

**Table 2:** Calibration function parameters during method validation by using LC-MS/MS.

Calibration curve number	Enalapril			Enalaprilat		
	*R* ^2^	Slope	Intercept	*R* ^2^	Slope	Intercept
1	0.9994	0.00679	0.00133	0.9996	0.00398	0.00104
2	0.9994	0.00666	0.00147	0.9996	0.00373	0.00175
3	0.9996	0.00694	0.00077	0.9994	0.00379	0.00137
4	0.9996	0.00695	0.00042	0.9998	0.00401	0.00097
5	0.9998	0.00697	0.00097	0.9998	0.00404	0.00120
6	1.0000	0.00712	0.00030	0.9990	0.00406	0.00129
Mean	0.9996	0.00691	0.00088	0.9995	0.00394	0.00127
STD	0.0002	0.00016	0.00047	0.0003	0.00014	0.00028
CV%	0.0234			0.0302		

**Table 3:** Absolute recovery of enalapril and enalaprilat from plasma (*n* = 6).

	Spiked concentration	Mean concentration	Recovery (%)
	(ng/ml)	(ng/ml)	
Enalapril	3	2.826	94.20
	100	103.62	103.62
	160	163.142	101.96

Enalaprilat	3	2.948	98.17
	100	108.401	108.40
	160	162.87	101.83

**Table 4:** Stability of enalapril under different storage conditions (*n* = 6).

Storage Condition	QC	Measured QC at	Mean ± SD	Accuracy	Stability
	(ng/ml)	0.0 h (ng/ml)	(ng/ml)	(%)	(%)
Autosampler stability 24 h at 4°C	3	2.886 ± 0.122	2.766 ± 0.126	93.03	95.83
	100	102.281 ± 4.214	100.579 ± 2.263	100.04	98.34
	160	170.216 ± 5.000	162.392 ± 9.214	105.09	95.40

Freeze and thaw stability at −20°C (3 cycles)	3	3.220 ± 0.117	3.106 ± 0.205	102.22	96.45
	100	106.009 ± 3.979	104.457 ± 2.687	107.96	98.54
	160	169.255 ± 3.668	17.110 ± 4.011		

Room temperature stability for 10 h	3	2.959 ± 0.065	2.946 ± 0.065	97.38	99.57
	100	99.061 ± 1.821	100.688 ± 3.356	101.32	101.64
	160	158.505 ± 6.208	164.31 ± 2.235	100.36	103.66

Accelerated stability at −30.0°C for 30 days	3	2.886 ± 0.122	2.823 ± 0.121	94.69	97.81
	100	102.281 ± 4.214	98.793 ± 3.235	99.53	96.59
	160	170.216 ± 5.000	144.847 ± 18.315	101.55	85.10

**Table 5:** Stability of enalaprilat under different storage conditions (*n* = 6).

Storage Condition	QC	Measured QC at	Mean ± SD	Accuracy	Stability
	(ng/ml)	0.0 h (ng/ml)	(ng/ml)	(%)	(%)
Autosampler stability 24 h at 4°C	3	2.948 ± 0.029	2.976 ± 0.035	99.19	100.94
	100	96.242 ± 0.994	96.172 ± 0.458	95.90	99.93
	160	148.473 ± 0.554	181.571 ± 0.553	92.72	102.09

Freeze and thaw stability at −20°C (3 cycles)	3	3.215 ± 0.063	3.099 ± 0.043	106.33	95.31
	100	105.243 ± 4.053	101.769 ± 2.538	103.29	96.70
	160	163.057 ± 2.557	167.983 ± 3.770	103.13	101.18

Room temperature stability for 10 h	3	3.130 ± 0.086	3.068 ± 0.023	102.97	98.02
	100	101.399 ± 2.769	99.845 ± 1.991	100.29	98.47
	160	157.842 ± 4.936	158.110 ± 4.021	97.71	100.17

Accelerated stability at −30.0°C for 30 days	3	2.948 ± 0.029	3.257 ± 0.088	102.99	110.47
	100	96.242 ± 0.994	99.723 ± 2.055	97.53	103.62
	160	148.473 ± 0.550	143.543 ± 2.118	92.25	96.68

**Table 6:** Pharmacokinetic parameters of healthy human volunteers with the test and reference formula (*n* = 30).

Calibration curve number	Enalapril 20 mg		Enalaprilat	
	Reference formulae (A)	Test formulae (B)	Reference formulae (A)	Test formulae (B)
*C*_max_ (ng/ml)	121.18 ± 50.23	114.42 ± 43.75	62.24 ± 22.14	57.67 ± 17.09
*T*_max_ (h)	0.897 ± 0.239	0.921 ± 0.308	3.539 ± 0.639	3.667 ± 0.592
AUC_0–72_ (ng·h/ml)	194.12 ± 73.94	186.22 ± 73.49	563.05 ± 149.09	550.34 ± 142.39
AUC_0–*∞*_ (ng·h/ml)	196.18 ± 74.10	189.79 ± 73.20	582.29 ± 152.18	571.31 ± 142.14
*k*el (h^−1^)	0.326 ± 0.208	0.338 ± 0.379	0.036 ± 0.009	0.034 ± 0.008
*t*1/2 (h)	3.04 ± 1.89	4.5 ± 3.68	20.67 ± 4.96	21.61 ± 5.27
Relative bioavailability (%) (B/A)	**94.417**			

*C*_max_: maximum plasma concentration, *t*_max_: time required to achieve maximum concentration, AUC_0–72_: area under the plasma concentration-time curve from time zero to 72 h, AUC_0−*∞*_: area under the plasma concentration-time curve from time zero to infinity, *K*el: elimination rate constant, and *t*1/2: elimination half- life.
